# Epithelioid Hemangioendothelioma: clinicopathologic, immunhistochemical, and molecular genetic analysis of 39 cases

**DOI:** 10.1186/1746-1596-9-131

**Published:** 2014-07-01

**Authors:** Uta Flucke, Rob JC Vogels, Nicolas de Saint Aubain Somerhausen, David H Creytens, Robert G Riedl, Joost M van Gorp, Anya N Milne, Clement J Huysentruyt, Marian AJ Verdijk, Monique M van Asseldonk, Albert JH Suurmeijer, Johannes Bras, Gabriele Palmedo, Patricia JTA Groenen, Thomas Mentzel

**Affiliations:** 1 Department of Pathology, Radboud University Medical Center, P.O. Box 9101, 6500 HB Nijmegen, The Netherlands; 2 Jules Bordet Institute, Brussels, Belgium; 3 Department of Pathology, Ghent University Hospital, Ghent, Belgium; 4 Department of Pathology, Maastricht University Medical Center, Maastricht, The Netherlands; 5 Department of Pathology, Diakonessenhuis Utrecht, Utrecht, The Netherlands; 6 Laboratory of Pathological Anatomy and Medical Microbiology (PAMM), Eindhoven, The Netherlands; 7 Department of Pathology, University Medical Center Groningen, University of Groningen, Groningen, The Netherlands; 8 Department of Pathology, Academic Medical Center, Amsterdam, The Netherlands; 9 Dermatopathology Bodensee, Friedrichshafen, Germany

**Keywords:** Epithelioid hemangioendothelioma, Vascular tumors, Soft tissue, Bone, Skin, Lung, Liver

## Abstract

**Abstract:**

**Background**

Epithelioid hemangioendothelioma is a malignant, often indolent vascular tumor which occurs at various anatomic sites. Based on a reciprocal translocation t (1;3)(p36;q25), a consistent *WWTR1-CAMTA1* fusion gene has been found. An alternate *YAP1-TFE3* fusion has been detected in a small and distinct subset of cases.

**Methods**

Thirty-nine tumors, from 24 females and 15 males with an age range 9–85 years, were located in soft tissue (head and neck [8], trunk [5], upper extremities [3], lower extremities [2], mediastinal [1], and paratesticular [1]), lymph node (1), breast (1), skin (2), bone (6), lung (7), and liver (2). The cases were investigated using a panel of immunohistochemical markers. The aforementioned fusion-genes were examined using RT-PCR and/or FISH in order to validate their diagnostic value.

**Results**

Follow-up available for 17 patients ranged from 3 months to 7 years (median interval 1.5 years). Eleven patients were alive without disease, 2 patients were alive with disease after 1.5 and 2 years, respectively. Four patients died of disease after 4 months (n = 1), 5 months (n = 2), and 1.5 years (n = 1).

The size, known for 30 lesions, was >3 cm in 9 of them. Histologically, all lesions had classical features, at least focally. Four tumors counted >3 mitoses/50 HPF.

Immunohistochemically, all cases tested stained positive for ERG (21), FLI1 (5) and CD31 (39). CD34 and D2-40 positivity was seen in 81% and 71% of the examined cases, respectively. 11/35 cases expressed pan-keratin and 6/20 cases CK8.18. TFE3 showed a nuclear reaction in 21/24 cases, irrespective of *TFE3* rearrangement.

Molecular genetically, 35/35 cases revealed one of the fusion genes by FISH and/or RT-PCR with *WWTR1-CAMTA1* in 33 cases and *YAP1-TFE3* in 2 cases.

**Conclusions**

These results demonstrate the high diagnostic value of FISH and RT-PCR in detecting the fusion genes of EHE. The immunohistochemical utility of TFE3 appears questionable in this study.

**Virtual Slides:**

The virtual slide(s) for this article can be found here: http://www.diagnosticpathology.diagnomx.eu/vs/4010279141259481

## Background

Epithelioid hemangioendothelioma (EHE), first described by Weiss and Enzinger in 1982, is a malignant vascular neoplasm with indolent behavior in the majority of cases [1]. Although, a progressive clinical course with tumor-related fatality has been documented in some instances, this lesion does not behave as aggressively as a conventional angiosarcoma [1-4].

EHEs arise in soft tissue, bone, skin and various parenchymatous organs [1-7].

The rarity of this tumor type, combined with the wide age distribution at presentation, the variety of anatomic sites and, in some cases, early metastases or multifocal disease leads to a wide range of differential diagnoses. This is complicated by the bland epithelioid cytomorphology and the presence of subtle, mostly intracytoplasmic, vascular lumina [1,2]. Classically, the vacuolated endothelial cells are arranged in short cords and strands recapitulating the primitive angiogenic cords of the yolk sac [8].

After discovering the nonrandom reciprocal t (1;3)(p36;q25) translocation [3], the corresponding fusion gene *WWTR1-CAMTA1* was detected [5,9]. More recently, an alternative gene fusion, *YAP1-TFE3,* was found in a small subset of lesions with distinct morphology and arising mainly in young patients [10].

In this study we have used a large cohort of cases from different anatomical sites to investigate the known fusion genes by fluorescent in situ hybridization (FISH, fusion probe) and reverse transcriptase-polymerase chain reaction (RT-PCR) in order to validate their diagnostic value. Furthermore, we have expanded immunohistochemical data with ERG, the most recently described antibody for endothelial differentiation, as well as TFE3, one of the known fusion proteins.

## Methods

The cases were retrieved from the (referral) files of the authors, and clinical details and follow-up were obtained from the referring physicians. Case 8 was already included in the series by Antonescu et al. [10]. The study was performed in accordance with the Code of Conduct of the Federation of Medical Scientific Societies in the Netherlands and Germany.

In all cases, the tissue was fixed in 4% buffered formalin, routinely processed and embedded in paraffin; 2–4 μm thick sections were stained with hematoxylin and eosin and immunohistochemically by the labelled Streptavidin Biotin technique using commercially available antibodies listed in Table 1. Appropriate positive and negative controls were used throughout.

**Table 1:** Details of used immunohistochemical antibodies

**Antibody**	**Clone**	**Dilution**	**Source**
ERG	EPR3864	1:2000	ABCam, Cambridge, UK
CD31	JC70 A	1:100	DAKO, Glostrup, Denmark
CD34	My10	1:100	BD Biosciences, Heidelberg, Germany
Pankeratin	AE1/3	1:50	DAKO, Glostrup, Denmark
CK8.18	CAM5.2	1:10	Becton Dickinson, San Jose, USA
D2-40	D2-40	1:100	DAKO, Glostrup, Denmark
EMA	Mc5	1:400	BioGenex, San Ramon, USA
ASMA	1A4	1:500	DAKO, Glostrup, Denmark
S-100	polyclonal	1:2000	DAKO, Glostrup, Denmark
Fli1	polyclonal	1:50	Santa Cruz Biotechnology, Heidelberg, Germany
TFE3	polyclonal	1:2000	Santa Cruz Biotechnology, Heidelberg, Germany
INI1	25/BAF47	1:50	BD Biosciences, Heidelberg, Germany

### Fluorescent in situ hybridization (FISH)

Interphase FISH was performed using a *WWTR1-CAMTA1* fusion probe (BACs RP11-1120, RP11-980). The red signal (rhodamine) flanked the distal region of *WWTR1* while the green signal (FITC) labeled the proximal region of the *CAMTA1* gene.

3 μM sections were deparaffinized with xylene and dried with ethanol after baking at 56°C for 16 hours. All tissue sections were pre-treated with a 30% solution of pre-treatment powder in 2xSSC and digested for 10 minutes with Proteinase K according to the instructions of the suppliers (MP Biomedicals Illkirch, France). After a second dehydrating step, the probes were applied to the sections and the covered slides were sealed with rubber cement, heat-denatured and hybridized at 37°C for 16 hours. Subsequently, all sections were counterstained with DAPI I in mounting medium (1000 ng/ml, Abbott, Wiesbaden, Germany) and visualized under a Zeiss Axioplan microscope using a HBO103 lamp and the appropriate filters for the three fluorescent dyes. A negative control was used in each case. A case was interpreted as positive when at least 10 of 50 counted tumor cells (20%) showed a (yellow) fusion signal.

### Reverse transcriptase-Polymerase chain reaction (RT-PCR)

RNA was extracted from formalin-fixed and paraffin-embedded tissues (FFPE) using RNA-Bee-RNA isolation reagent (Bio-Connect BV, Huissen, the Netherlands) according to standard procedures. RNA quantity and quality were determined by NanoDrop measurement (Fisher Scientific, Landsmeer, the Netherlands) and, subsequently, cDNA synthesis was performed using Superscript II (Invitrogen Life Technologies Europe, Bleiswijk, the Netherlands) and random hexamers (Promega Nederland, Leiden, the Netherlands).

The cDNA was tested by the reverse transcription-polymerase chain reaction (RT-PCR) for the *HMBS* (hydroxymethylbilase synthase) housekeeping gene using the primers forw150 5’-TGCCAGAGAAGAGTGTGGTG-3’, rev150 5’-ATGATGGCACTGAACTCCTG-3’, forw250 5’- CTGGTAACGGCAATGCGGCT-3’, rev250 5’- TTCTTCTCCAGGGCATGTTC-3’.

For detection of the t (1;3) (p26.3;q25) translocation, the following primers were used: *WWTR1* (NM_001168278.1) forward primers in exon3 5’-GCTGGGAGATGACCTTCACGGC-3’ and exon4 5’-CCGTCAGTTCCACACCAGTGCCTC-3’ and *CAMTA1* (NM_015215.2) reverse primers in exon8 rev 5’-GGCTGGGGCTTGGTCTGGTG-3’ and, because of the use of FFPE tissues with suboptimal RNA/cDNA quality, multiple exon9 primers were used: (1) exon9 rev 5’-GCGAGATGATGCGGTGTTTGGC-3’, (2) exon 9 rev 5’ CTCGGTGCTGCTCTGGTGCAG-3’, (3) exon 9 rev 5’- CACCGGGCTGTCCACCATGTC-3’ and (4) exon 9 rev 5’-GGACAGGCTCTCCGAGCTGCC-3’.

For the detection of the *YAP1-TFE3* fusion, the primers *YAP1* (NM_001130145.2) exon1 forw 5’- CTCCGGAAGCTGCCCGACTCC-3’ and *TFE3* (NM_006521.4) exon4 rev 5’-GAGTGTGGTGGACAGGTACTG-3, *TFE3* exon 6 rev 5’- GTTGCTGACAGTGATGGCTGG-3’, *TFR3* exon 8 rev 5’-CGGGTCACTGGACTTAGGGATGAGA-3’ and *TFE3* exon 10 rev 5’- CCTGCCCTCCTCCTCAATGTCC-3’ were used. The PCR products were analyzed by agarose gel electrophoresis. The sequence of the differently sized PCR-products was obtained by Sanger sequencing and confirmed the presence of the fusion gene.

Negative controls for RT-PCR were 2 epithelioid hemangiomas, 1 epithelioid fibrous histiocytoma, 1 angiosarcoma, 1 epithelioid sarcoma-like/pseudomyogenic hemangioendothelioma and 1 soft tissue angiofibroma.

## Results

### Clinical data

Clinical data are presented in Table 2.

**Table 2:** Clinical data

**Case**	**Sex/Age**	**Site**	**Size (cm)**	**Therapy**	**Rec/Met, m**	**Follow-up**
1	f/42y	toe	2.0	E	NA	NA
2	f/63y	occipital	1.0	WE, RT, ME	cerv LN, 14	NED, 2y
3	f/58y	groin	NA	NA	NA	NA
4	f/66y	lung (mf)	NA	NA	NA	NA
5	m/69y	oral cavity	0.8	WE	-	NED, 2y
6	m/42y	groin	3.6	NA	NA	NA
7	m/10y	nose bridge	1.1	NA	NA	NA
8	m/14y	LN, groin	2.0	WE	NA	NA
9	f/70y	heel	1.1	NA	NA	NA
10	m/61y	skin/finger	0.5	WE	-	NED, 2y
11	f/71y	neck	1.1	E, R1	-	NED, 7y
12	m/51y	thoracic spine	NA	NA	NA	NA
13	f/30y	upper arm	1.9	NA	NA	NA
14	m/70y	lower arm	2.0	NA	NA	NA
15	f/85y	paravertebral	5.4	NA	NA	NA
16	f/78y	breast	NA	NA	NA	NA
17	f/41y	neck	2.5	WE, RT	-	NED, 3m
18	f/60y	axilla	4.5	E, RT	-	NED, 1y
19	m/68y	lung	1.4	WE, TKI*	lung, pleura, mediastinum, 13	AWD, 2y
20	m/54y	lung	2.2	WE	NA	NA
21	m/43y	mediastinum	7.5	E	NA	NA
22	f/46y	liver (mf)	4.5	WE	-	NED, 1y
23	f/70y	liver	NA	CT	pleura, 0	AWD, 1.5y
24	m/49y	maxilla	3.1	WE, RT,ME	cerv LN, 5	NED, 1y
25	f/26y	humerus	NA	E	-	NED, 5y
26	m/10y	paratesticular	1.4	E	NA	NA
27	f/55y	rib	4.5	WE	NA	NA
28	f/49y	mandible	2.5	WE	cerv LN	NA
29	f/37y	neck	3.0	E	LN,bone,lung	DOD, 4 m
30	f/81y	femur	NA	NA	NA	NA
31	m/19y	lung (mf)	NA	NA	NA	NA
32	f/41y	groin	1.8	WE, RT	Vulva	NED, 1y
33	f/77y	pubic skin	2.5	WE	-	NED, 3y
34	m/59y	lung	3.0	WE, ME	pleura, bone, mesentery, 3	DOD, 1.5y
35	f/25y	neck	7.5	E	cerv LN, 0	NA
36	f/9y	lung (mf)	NA	CT	pleura	DOD, 0.5y
37	f/10y	lung (mf)	11	CT, RT	chest wall	DOD, 0.5y
38	m/51y	elbow	2.4	E	NA	NA
39	f/75y	neck	2.5	WE	NA	NA

*Rec*, Recurrence; *Met*, Metastases; *m*, Months; *y*, Year(s); *E*, Excision; *WE*, Wide excision; *mf*, Multifocal; *NED*, No evidence of disease; *AWD*, Alive with disease; *DOD*, death of disease; *RT*, Radiotherapy; *CT*, Chemotherapy; *ME*, Metastasectomy; *NA*, Not available; *cerv*, Cervical; *LN*, Lymph node; int jug vein, internal jugular vein; TKI*, Tyrosine kinase inhibitor pazopanib.

Of the 39 patients, 24 were females and 15 were males. The age ranged from 9 to 85 years (mean 50 years; median 51 years). Anatomical sites of soft tissue lesions were as follows: head and neck region (8), trunk (5), upper extremities (3), lower extremities (2), mediastinal (1) and paratesticular (1). One tumor was located in an inguinal lymph node, one in the breast, and 2 lesions in the skin (finger, pubic region). Six neoplasms arose in the bone and 7 in the lung with multifocal disease in 4 cases. Two tumors were situated in the liver. One of them showed multifocality. Metastatic disease was diagnosed in 11/19 cases with occurrence in lymph nodes (Cases 2, 24, 28, 35), lung (Cases 19 and 29), pleura (Cases 19, 23, 34, 36), bone (Cases 29 and 34), chest wall (Case 37), mediastinum (Case 19), mesentery (Case 34), and vulva (Case 32). Three patients had metastases to multiple sites (Cases 19, 29, 34).

All but 3 tumors were treated by surgery with wide excision in 15 cases and marginal excision in 9 cases. Additional radiotherapy was applied in 5 cases and metastasectomy was performed in Cases 2, 24 (cervical lymph node) and 34 (mesentery). One patient (Case 19) received pazopanib when metastases and progression were obvious. Two patients were treated by chemotherapy only (Cases 23 and 36). Another patient with multifocal lung disease received radio-chemotherapy (Case 37).

Follow-up information, available for 17 patients, ranged from 3 months to 7 years (median interval 1.5 years). Eleven patients were alive without disease, 2 patients were alive with disease after 1.5 and 2 years, respectively. Four patients died of disease after 4 months (n = 1), 5 months (n = 2), and 1.5 years (n=1). Two of them were children.

The patient from Case 8 had a diagnosis of Langerhans cell histiocytosis 11 years prior to the EHE. Case 19 was known with a previously excised cutaneous leiomyosarcoma.

### Gross findings

Tumor size ranged from 0.5 to 11 cm (mean 3 cm). The cut surface appeared solid, white-tan and showed some hemorrhage in a number of cases.

### Histological findings

**Figure 1:**
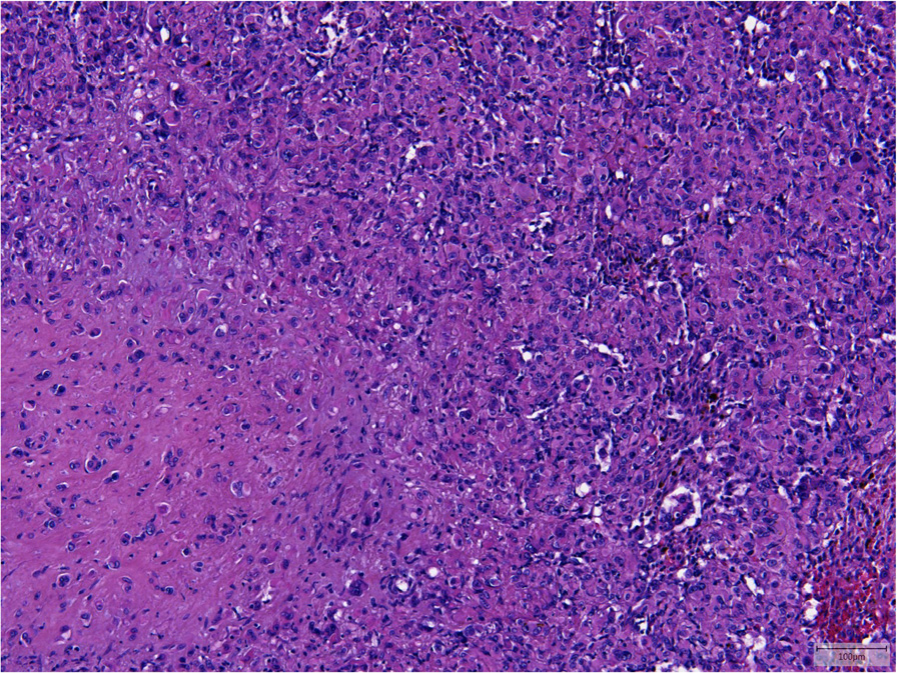
Case 6 showed solid areas without a myxohyaline stroma in addition to the classical morphological features (lower left).

**Figure 2:**
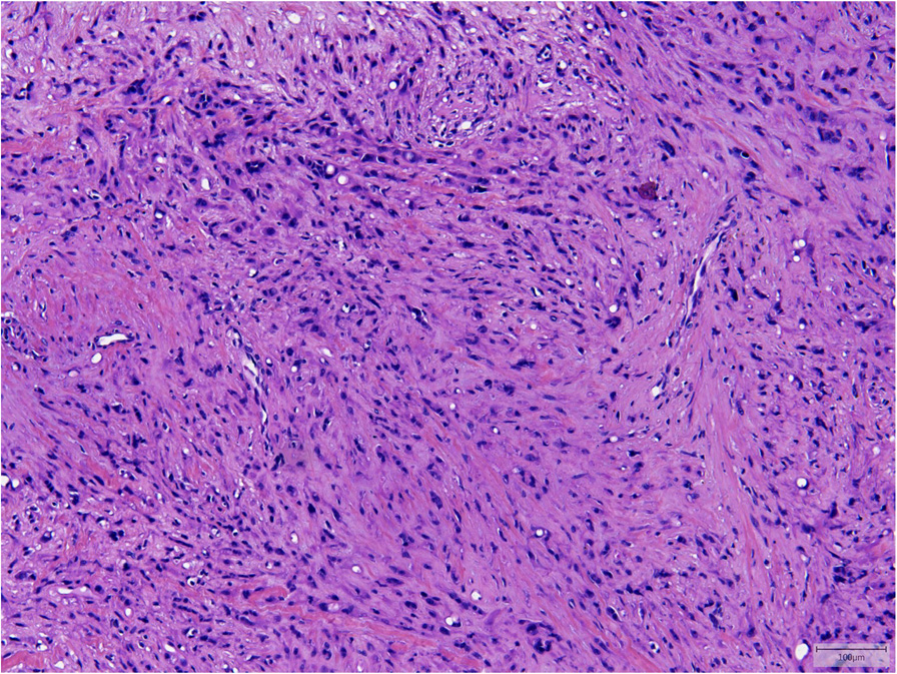
Striking nuclear atypia was seen in approximately 30% of the cases (Case 14).

**Figure 3:**
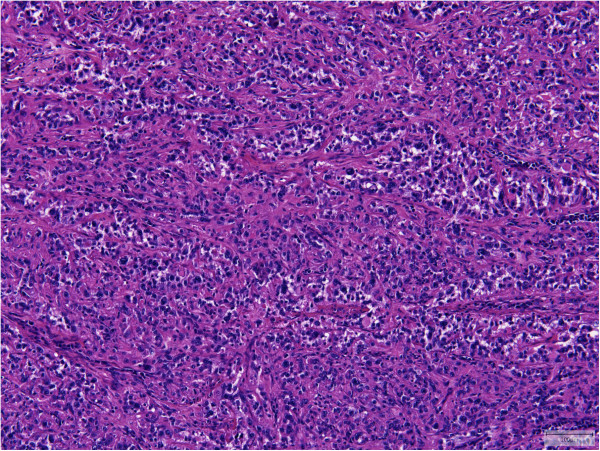
**More vasoformative structures and nuclear atypia was present in Case 8 which harbored a *YAP1-TFE3 *** **fusion.**

All tumors showed an infiltrative growth with relatively sharp demarcation in 13 cases. Multinodularity was observed in 10 cases with separate nodules in some of these cases. Vasculocentric growth was present in 15 tumors with occasional intravascular expansion and occlusion of larger vessels. Dabska-like intravascular projections were seen in one case (Case 30). Perineural invasion was found in 5 tumors (Cases 2, 3, 10, 11, 13).All lesions were composed of epithelioid cells arranged in strands, cords and nests. There was also often a histiocytoid cellular appearance. Additional fusiform cells were seen in 20 cases. Solid highly cellular areas were encountered in 10 cases (Cases 6, 9, 15, 17, 21, 30, 34, 35, 37, 39) (Figure 1). The nuclei were commonly vesicular with small often distinct nucleoli. Mild nuclear atypia was at least focally observed in 16 lesions and striking nuclear atypia in another 12 cases (Cases 6, 8, 11, 12, 14, 15, 17, 24, 25, 32, 38, 39) (Figures 2 and 3). Mitoses ranged from 0 to 22/50 HPF. The 2 tumors with the highest mitotic index showed respectively 12 and 22 mitotic figures/50 HPF (Cases 30 and 34). Two lesions showed 4 mitotic figures/50 HPF (Cases 32 and 37) and all other lesions did not exceed 3 mitoses/50 HPF. Nuclear pseudoinclusions were seen in the cases with increased atypia. All lesions showed an abundant hyaline cytoplasm with variable intracytoplasmic vacuoles. More or less well-formed multicellular vascular channels were present in 11 lesions and this was a prominent feature in Case 8 (Figure 3). Some lumina were occluded by hyaline thrombi. A myxohyaline stroma was at least focally present in all cases and some lesions also induced a desmoplastic reaction. Stroma was absent in highly cellular areas. Fifteen neoplasms had (ischemic) necrosis, and calcification was seen in 2 lesions (Cases 9 and 10). One tumor contained metaplastic bone (Case 15). Multinucleated giant cells were scattered throughout the lesion in one case (Case 39). Hemosiderin deposition and a predominantly marginal inflammatory reaction were seen in some cases.

According to the criteria by Deyrup et al. [4], 12 tumors were classified as high risk with a tumor size >3 cm in 9 lesions and a high mitotic rate (>3/50 HPF) in 4 instances including one tumor which showed both. Of the 6 cases with follow-up, 2 patients died of disease (Cases 34 and 37; lung lesions) and 4 were alive without evidence of disease (Cases 18, 22, 24, 32), 2 of them after metastasectomy (lymph node, vulva). In 8 of the 19 cases fulfilling low risk criteria, follow-up was available. Six patients (Cases 2, 5, 10, 11, 17, 33) showed no evidence of disease (one after metastasectomy), while one was alive with metastatic disease (Case 19) and one died of disease (Case 29).

### Immunohistochemical findings

The results are shown in Table 3.

**Table 3:** Immunohistochemical analyses

**Case**	**ERG**	**CD31**	**CD34**	**panCK**	**CK8.18**	**EMA**	**TFE**	**Others**
1	+	+	f+	+	-	ND	ND	D2-40 f+
2	ND	+	f+	-	ND	-	+	S100 -, SMA -
3	ND	+	-	-	ND	-	f+	S100 -
4	ND	+	-	-	ND	-	-	S100 -, SMA -
5	ND	+	ND	-	ND	ND	+	S100 -
6	ND	+	+	-	ND	ND	f+	SMA -, INI1 +
7	ND	+	+	-	ND	ND	+	S100 f+
8	+	+	ND	ND	ND	ND	+	SMA -
9	ND	+	ND	ND	ND	ND	f+	SMA -
10	ND	+	f+	-	ND	-	f+	S100 f+, INI1+
11	ND	+	+	-	ND	ND	f+	Fli1 +, D2-40 -
12	ND	+	+	-	ND	ND	-	Fli1 +
13	ND	+	ND	f+	ND	ND	+	
14	ND	+	+	ND	ND	ND	-	Fli1 +
15	ND	+	ND	ND	ND	ND	f+	Fli1 +
16	ND	+	ND	-	ND	ND	+	D2-40 +
17	+	+	+	f+	-	-	f+	S100 -, SMA -
18	+	+	+	-	-	ND	+	
19	+	+	+	f+	f+	-	f+	D2-40 +
20	+	+	+	-	-	ND	f+	
21	+	+	ND	-	ND	ND	ND	S100 -
22	+	+	+	-	f+	ND	f+	
23	ND	+	+	+	+	f+	ND	D2-40+
24	ND	+	-	f+	f+	-	f+	SMA f+
25	ND	+	+	-	-	ND	ND	S100 -
26	+	+	f+	-	-	ND	ND	D2-40 -, SMA -
27	+	+	f+	-	-	ND	ND	
28	+	+	f+	-	-	ND	ND	
29	+	+	-	-	-	-	ND	S100 -
30	+	+	-	-	-	-	ND	SMA -
31	+	+	+	-	ND	ND	f+	
32	+	+	+	f+	f+	ND	ND	S100 -
33	+	+	+	-	-	ND	ND	
34	+	+	+	f+	f+	ND	ND	
35	ND	+	-	f+	ND	-	ND	Fli1+, INI1 +,
36	+	+	+	-	-	ND	ND	
37	+	+	+	f+	-	ND	f+	
38	+	+	ND	-	ND	ND	ND	SMA -
39	+	f+	+	+	-	ND	+	D2-40 f+
%+	100	100	81	31	30	9	88	

ND, not done; f, focal expression.

**Figure 4:**
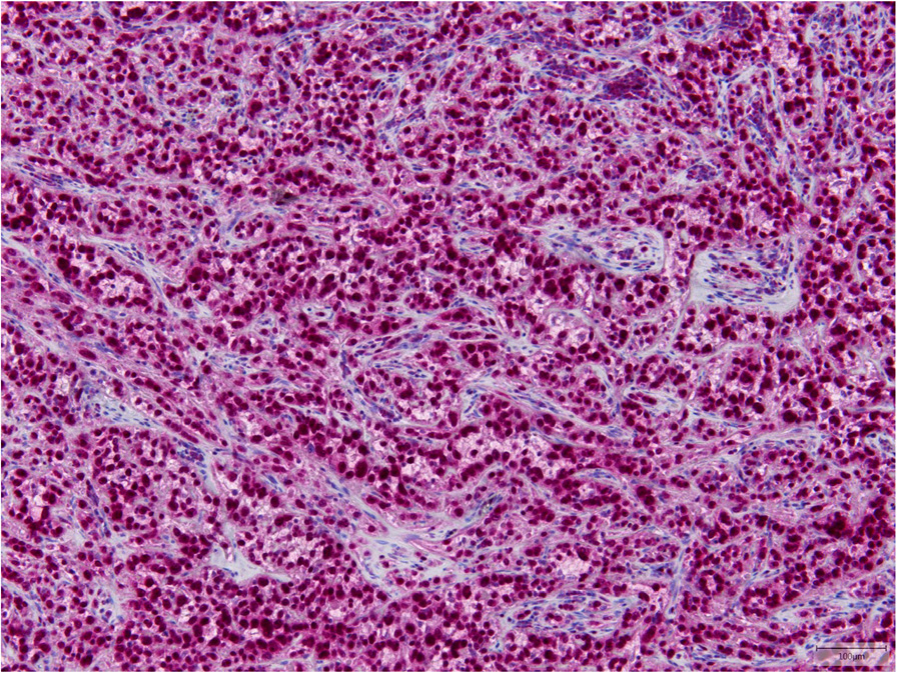
ERG was positive in all cases tested.

**Figure 5:**
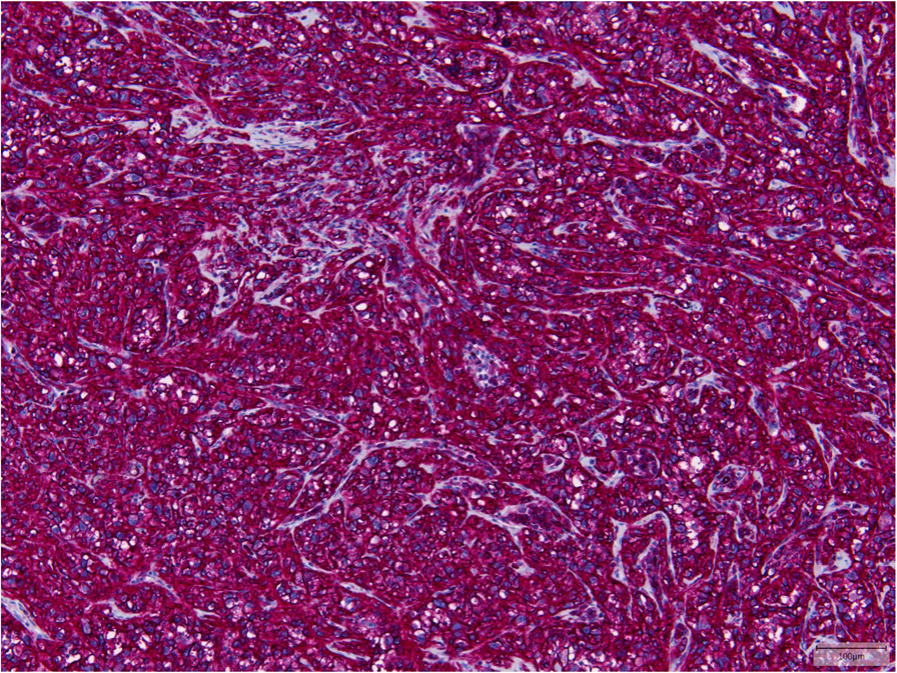
CD31 was strongly expressed in 38/39 cases.

**Figure 6:**
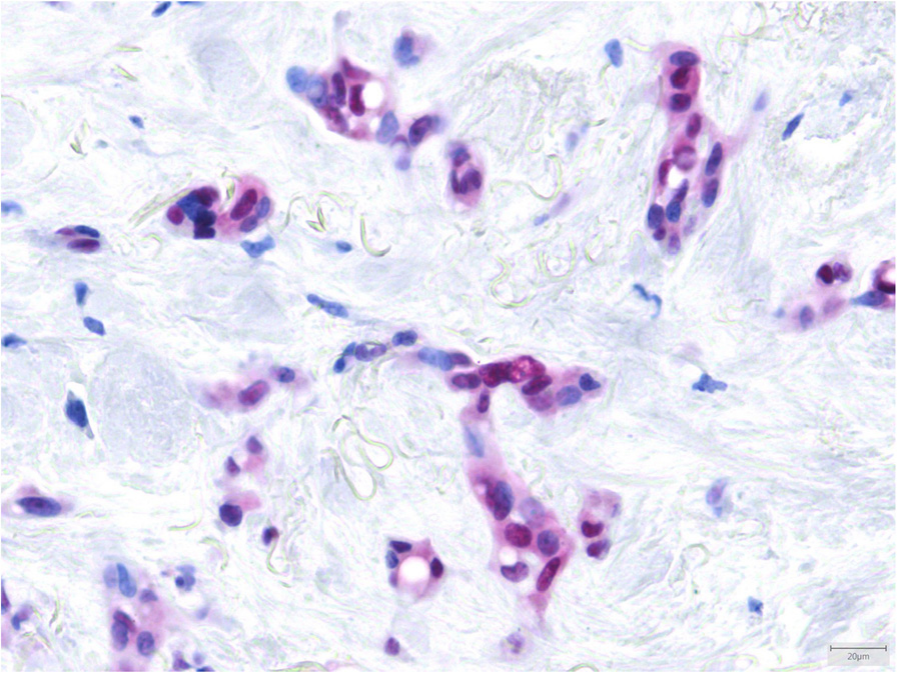
**TFE3 showed a nuclear reaction in cases with a *WWTR1-CAMTA1 *** **fusion.**

21/21 cases were positive for ERG (Figure 4). A membranous reaction for CD31 was demonstrated in all 39 cases (Figure 5) with a focal expression in one case. CD34 staining was seen in 25/31 (81%) cases with incomplete reaction in 6 samples. FLI1, performed in 5 cases, showed a positive result in all 5 cases. D2-40 was positive in 5/7 cases (71%). Two of these cases had a focal staining pattern. 11/35 lesions (31%) stained positive for pan-keratin with a focal reaction in 8 tumors. CK8.18 was focally immunoreactive in 5/20 cases and strongly reactive in another case (30%). EMA was negative in 10/11 cases. The positive case showed patchy staining (9%). ASMA was focally expressed in 1/10 lesions (10%). S-100, which was performed in 11 cases, was patchy positive in 2 of them (18%). INI1 was retained in all 3 samples tested. TFE3, examined in 24 cases, showed a nuclear reaction, at least focally, in 21 cases (88%) (Figure 6), including the two *TFE3*-rearranged neoplasms (Cases 8 and 31).

### Molecular genetic findings

Molecular genetic results are presented in Tables 4 and 5.

**Table 4:** Molecular analyses

**Case**	***WWTR1-CAMTA1***	***WWTR1-CAMTA1***	***YAP1-TFE3***
	**RT-PCR**	**FISH**	**RT-PCR**
1	X	X	X
2	ex4-ex9	X	ND
3	X	X	X
4	ex4-ex8	X	ND
5	ex4-ex8	Neg	ND
6	X	Pos	X
7	Neg	Pos	Neg
8	Neg	Neg	ex1-ex4
9	ex4-ex8	Pos	ND
10	ex4-ex8	Pos	Neg
11	ex4-ex9	Pos	ND
12	ex4-ex9	X	Neg
13	ex4-ex8	Pos	ND
14	Neg	Pos	Neg
15	ex4-ex8	X	ND
16	ex4-ex8	X	ND
17	ex4-ex8	X	ND
18	ex4-ex8	Neg	ND
19	ex4-ex9	Pos	Neg
20	ex4-ex9	Pos	ND
21	Neg	Pos	Neg
22	ex4-ex9	Pos	Neg
23	ex4-ex8	X	ND
24	ex4-ex8	Pos	ND
25	ex3-ex9	Pos	Neg
26	X	Pos	X
27	ex4-ex8	Pos	ND
28	X	Pos	X
29	X	X	X
30	ex4-ex8	Pos	ND
31	Neg	ND	ex1-ex4
32	X	Pos	X
33	X	Pos	X
34	ex3-ex9	Pos	Neg
35	ex4-ex9	Pos	ND
36	ex3-ex9	X	Neg
37	X	Pos	X
38	X	X	X
39	X	Pos	X
	23 cases Pos	23 cases Pos	2 cases Pos

Ex, exon; neg, negative; pos, positive; x, failed; ND, not done.

**Table 5:** Fusion-transcripts for both fusion genes

***WWTR1-CAMTA1***	**Number of cases**
exon 4-exon 8	13
exon 4-exon 9	7
exon 3-exon 9	3
***YAP1-TFE3***	**Number of cases**
exon 1-exon 4	2

The analysis failed for both methods in 4 out of 39 cases (Cases 1, 3, 29, 38). The remaining 35 cases revealed one of the known fusion genes with *WWTR1-CAMTA1* positivity in 33 cases (94%) and *YAP1-TFE3* in 2 instances (6%).

**Figure 7:**
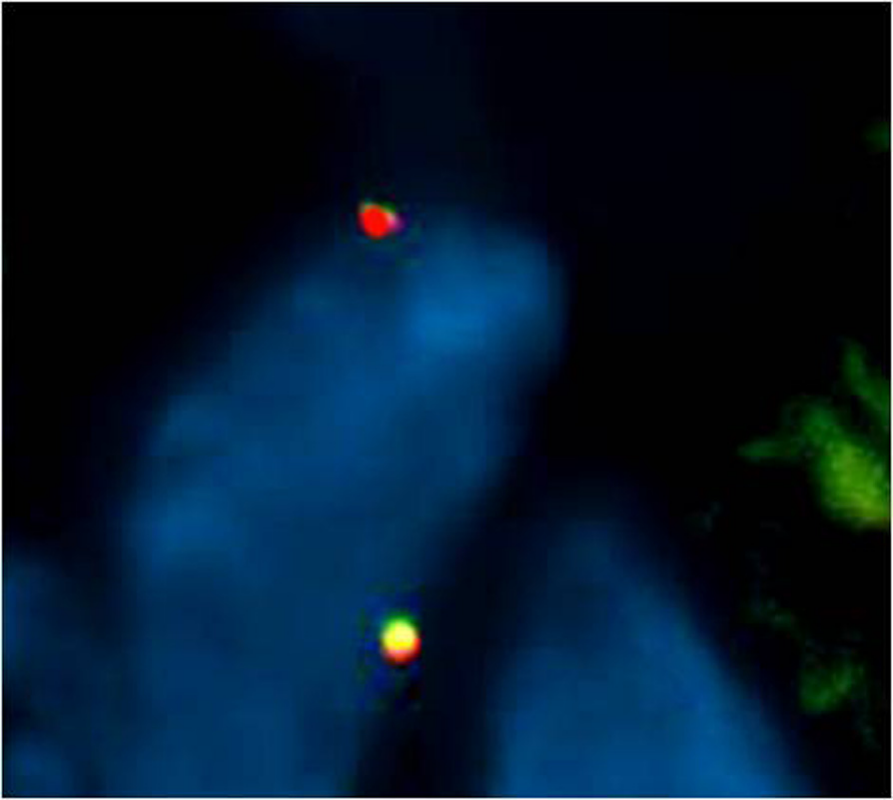
**FISH identified a fusion of *WWTR1-CAMTA1.***

In the FISH analysis (fusion probe), 23 out of 38 cases tested, were positive for *WWTR1-CAMTA1* (Figure 7) and 3 were negative (including one with a *YAP1-TFE3* fusion). 12 samples showed no hybridization result probably due to the poor DNA quality.

Using RT-PCR, 25/28 cases were detected to harbor a fusion gene; 23 were *WWTR1-CAMTA1* positive and 2 for *YAP1-TFE3*. Eleven cases were not evaluable because of the poor RNA quality. Seven of these cases were positive for *WWTR1-CAMTA1* with FISH. In other words, 8 cases with a detected fusion gene via RT-PCR showed no signal with FISH. Additionally, 3 cases which were negative with RT-PCR had a positive FISH result (*WWTR1-CAMTA1*).

**Figure 8:**
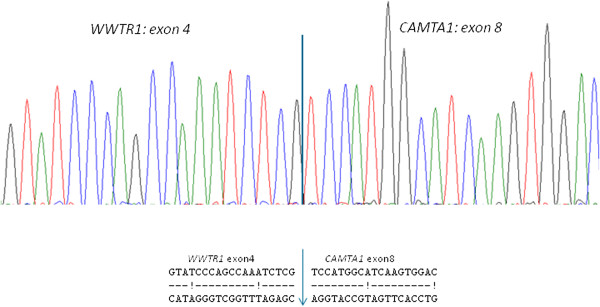
**Sequencing of the RT-PCR product shows an in-frame fusion between exon 4 of *WWTR1 *** **and exon 8 of *CAMTA1 *** **in the chimeric transcript in 13 cases.**

Several fusion-transcript variants for *WWTR1-CAMTA1* were found: exon 4-exon 8 in 13 cases (Figure 8), exon 4-exon 9 in 7 cases and exon 3-exon 9 in 3 cases.

**Figure 9:**
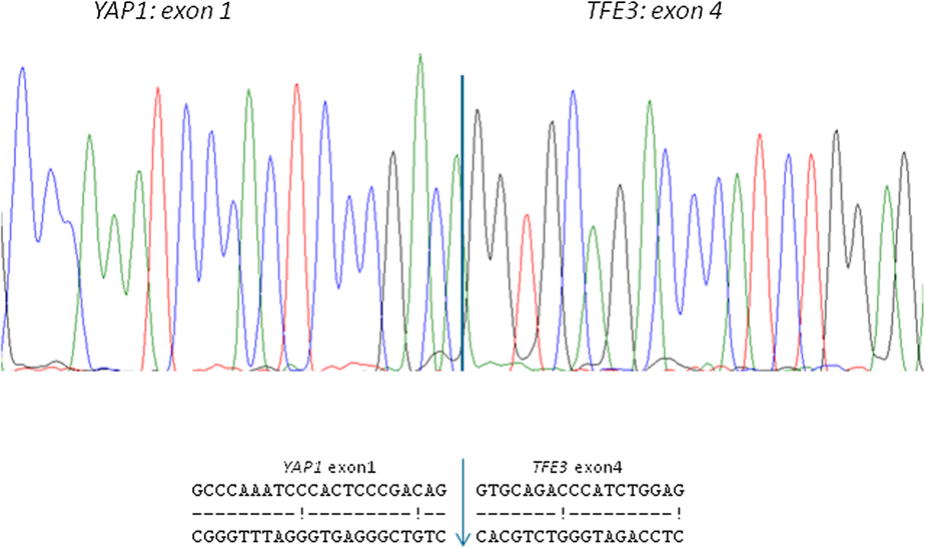
***YAP1 *** **exon1 was fused to *TFE3 *** **exon 4 in two cases (ABI sequence).**

The fusion-transcript for *YAP1-TFE3* were exon 1-exon 4 in both cases (Figure 9).

## Discussion

Similar to other translocation associated tumors, the t (1;3)(p36;q23-25) or t(11;X)(q13;p11) translocations are seemingly early causative events in EHE oncogenesis [5,11] which initiate a novel transcription program in cells with endothelial properties [5].

The corresponding fusion proteins, WWTR1-CAMTA1 and YAP1-TFE3, may serve as chimeric transcription factors, which manifest their oncogenic function via a promoter switch [5,12]. Another possible oncogenic effect could be due to the loss of regulatory domains of the C-terminus of *WWTR1* or *YAP1* and the N-terminus of *CAMTA1* or *TFE3*[5].

*WWTR1* on 3q23-24, encodes a transcriptional co-activator involved in mesenchymal stem cell differentiation and is highly expressed in endothelial cells [5,12], while CAMTA1, a calmodulin-binding transcription activator, located on 1p36.23, has been proposed as an oncogene under the control of the *WWTR1* promotor. The latter is supported by the occurrence of an in-frame fusion of the C terminus of *CAMTA1* to *WWTR1,* arguing against loss of function [5].

Alternatively, the *YAP1-TFE3* in-frame fusion was recently found in a small and distinct subset of EHE [10].

*YAP1,* located on 11q13, is a member of the *FAT*-family genes similar to *WWTR1,* and encodes for another WW-domain containing transcriptional co-activator [10]. The protein of *TFE3*, located on Xp11.22, is a member of the microphtalmia transcription factor family with oncogenic properties in several tumor types [10,13].

Although these gene fusions have not been identified in other neoplasms, in particular other epithelioid vascular tumors, it is not clear yet whether they are unique to this entity [5,9].

Using both, FISH and RT-PCR, we detected a *WWTR1-CAMTA1* fusion in 23 cases with discrepancies in 5 cases whereby the results were positive with one method only (and negative for the other). This could be due to the primers used missing potentially new breakpoints (RT-PCR) and possibly a low number of tumor cells showing a fusion signal by FISH.

The known *WWTR1* breakpoints are in intron 3 and 4, and the *CAMTA1* breakpoints are located in intron 7 and 8, and at different sites within exon 9 [5,9]. The resulting fusion-transcript variants which we found in our study were exon 4-exon 8 (n = 13), exon 4-exon 9 (n = 7) and exon 3-exon 9 (n = 3) (listed in decreasing frequency).

Two cases (6%) revealed the recently detected fusion *YAP1-TFE3* by RT-PCR*.* One of them (Case 8) was included in the study by Antonescu et al. [10], in which the gene fusion was demonstrated by FISH. This case was not only remarkable because of the histomorphological characteristics as reported in the mentioned paper [10] but also because it occured in a lymph node as the primary site. A more frequently occurring metastasis should be excluded in such cases [2,14]. In contrast, the other case with a *YAP1-TFE3* fusion showed a classical morphology of a multifocal lung EHE (Case 31). Interestingly, these 2 cases involved adolescents/young adults in concert with the finding of young age reported by Antonescu et al. [10].

*TFE3* rearrangements are also present in alveolar soft part sarcoma, certain pediatric renal cancers and a subset of PEComas [15-18]. The use of an antibody against the C-terminal portion of TFE3 seems to be a useful diagnostic tool in all rearranged tumors [10,19], but one has to be aware of an unspecific staining pattern [17] as we found in a proportion of cases with *WWTR1-CAMTA1* fusion.

ERG and FLI1 are transcription factors of the ETS-family which are expressed in endothelial cells. In addition to CD31, these 2 markers are helpful in highlighting the vascular nature of EHE [10,20,21] and showed expression in all cases examined. These markers are of course expressed in all other vascular lesions [8,20,21], therefore, detection of the aforementioned fusion genes may be a valuable discriminatory tool in diagnosing EHE, especially in difficult cases.

In 2008, a proposal for risk stratification was made based on clinicopathological features of 49 soft tissue EHEs. It seems that higher mitotic activity (>3/50 HPF) and tumor size exceeding 3 cm are associated with higher mortality, irrespective of anatomic site, presence of cytological atypia, tumor cell spindling, or necrosis [4]. Due to the small number of our cases with available clinical data, we were able to show only tendencies for the low-risk and high-risk category. Furthermore, in skin lesions, a favorable outcome is well known similar to all other sarcomas at this site [2]. Whether it is useful to include lung-, liver- and bone lesions in this risk stratification scheme remains uncertain so far, especially with respect to multifocal occurrence (or early metastases) [9].

Differential diagnoses of EHE depend on anatomic site and age. Carcinomas, myoepithelial tumors, epithelioid sarcoma, mesothelioma, extraskeletal myxoid chondrosarcoma, myxoid liposarcoma and especially other vascular tumors with epithelioid morphology, such as epithelioid hemangioma, cutaneous epithelioid angiomatous nodule, epithelioid angiosarcoma and pseudomyogenic hemangioendothelioma (epithelioid sarcoma-like hemangioendothelioma, PM-H) can enter the differential diagnoses [1,2,8,22-24]. In bone, chondrosarcoma can also be a potential pitfall.

Epithelioid hemangioma occurring in skin, soft tissue and bone is distinguished by a lobular architecture of well-formed vessels with a pericytic cuff, highlighted by ASMA/MSA (muscle specific actin) immunohistochemical reaction [1,22,25,26]. In the center of the lesion, the plump epithelioid cells are sometimes rather tightly packed and arranged in solid sheets simulating a more aggressive tumor. The distinctive zonation pattern with peripheral maturation is a helpful finding [25,26]. A myxohyaline stroma is not a feature of epithelioid hemangioma. As in EHE, vascular invasion can occur. Multifocality of epithelioid hemangioma with involvement of soft tissue and/or bone, skin and lymph nodes should not be confused with metastases of EHE [26,27].

Cutaneous epithelioid angiomatous nodule is a circumscribed lesion composed of a sheet-like proliferation of epithelioid cells with eosinophilic cytoplasm. Intracytoplasmic vacuoles are numerous and mitotic figures can be present. This lesion lacks cords and strands of tumor cells in a myxohyaline or fibrous stroma which is seen in EHE [22].

Epithelioid cells with eosinophilic cytoplasm are present to a variable extent in approximately 70% of angiosarcomas (AS) of soft tissue [23] and, of course, they can be prominent in AS at other sites as bone, skin and parenchymatous organs. There is considerable nuclear pleomorphism present and a high mitotic index, which are not typical features of EHE. Lumen formation ranges from irregular vascular channels to intracytoplasmic vacuoles or when absent there can be a diffuse, nest- or sheet-like growth pattern. The hemorrhagic background seen in angiosarcoma is not characteristic for EHE. Keratins can be expressed in both EHE and angiosarcomas (in ca. 30% of the cases, respectively) [23].

Pseudomyogenic hemangioendothelioma (epithelioid sarcoma-like hemangioendothelioma, PM-H) is composed of loose fascicles and sheets of plump spindle cells and/or epithelioid cells with vesicular nuclei and bright eosinophilic cytoplasm. A myxoid background rarely exists in PM-H and (intracytoplasmic) vascular formations are described as more elusive. Angiocentric growth may occur in both entities. Expression of keratins is an overlapping feature, although CD34 and pan-keratin MNF116 are lacking in PM-H [8,24]. Recently, *SERPINE1-FOSB*, has been identified as a specific fusion gene in PM-H [28].

Carcinomas tend to manifest as more pleomorphic, mitotically active tumors [1] evoking a desmoplastic response and usually exhibit stronger keratin expression whereas endothelial markers are absent.

Myoepithelial tumors are composed of variable proportions of epithelioid, spindle, clear and/or plasmocytoid cells. However, cords and strands embedded in a (chondro) myxoid/hyaline matrix is a shared feature and cytoplasmic vacuolation can occur [29]. Endothelial markers are absent in myoepithelial tumors. S-100 and keratins, usually expressed in myoepithelial tumors [29], can also be occasionally positive in EHE as demonstrated in two of our cases [2]. *EWSR1* and *PLAG* rearrangements are characteristic genetic changes in myoepithelial tumors [30-33].

Extraskeletal myxoid chondrosarcoma shows a multinodular architecture. The monomorphous small round or short spindle cells are loosely arranged and show delicate anastomosing strands distributed in a prominent myxoid stroma. Although some vacuolated cells can be observed, the cytoplasm is more eosinophilic [1,34] and vascular markers are not expressed. *NR4A3* rearrangement is a pathognomonic genetic aberration [35].

Conventional epithelioid sarcoma (ES) tends to grow in confluent nodules with central necrosis or hyalinization. The epithelioid to spindled cells show deeply eosinophilic cytoplasm. Vacuolated cells can possibly be confused with intracytoplasmic lumina of EHE [36]. When the matrix is myxoid, there are often still prominent collagen bundles [37], a feature which is not seen in the myxohyaline stroma of EHE. Immunohistochemically, this tumor type typically shows keratin- and EMA-expression, loss of INI1 [38] and occasionally a membranous CD31 staining [39]. CD34 and D2-40 can be either positive or negative in both tumor types [40-42]. Interestingly, ERG and FLI1 (antibody to the N-terminus) expression was recently shown in up to 70% and more than 90% of ES, respectively [40,41]. Therefore, these markers can only be interpreted in the right context together with CD31 and INI1. The latter is retained in EHE [38] as shown in some of our cases.

Myxoid liposarcoma shows a prominent myxoid matrix with enhanced cellularity at the periphery of the tumor nodules. The primitive cells are uniformly round to oval-shaped. Small signet ring lipoblasts could possibly be confused with the vacuolated cells of EHE, but there is no abundant pale-eosinophilic cytoplasm in a myxoid liposarcoma. The delicate arborizing vasculature in myxoid liposarcoma is very characteristic. When genetics are taken into account, DDIT3 is the consistent fusion partner in this lesion [42].

As well as the aforementioned vascular lesions, chondrosarcoma is an important differential diagnosis in primary bone tumors and shows uniform atypical chondroblasts suspended in an extensive myxoid matrix. Cytoplasmic vacuolation can be misinterpreted but the nuclei in chondrosarcoma are hyperchromatic and the scant cytoplasm is eosinophilic [42]. Vascular immunohistochemical markers are absent.

Epithelioid mesothelioma could be a relevant differential diagnosis at appropriate sites because of the abundant, sometimes vacuolated cytoplasm of mesothelial cells and the possible myxoid stroma. Although pankeratin and D2-40 can be positive in both malignancies, as also demonstrated in a number of our cases, calretinin, keratin 5/6, keratin 7, and WT1 are typically distinguishing mesotheliomas [43].

## Conclusions

The identification of the fusion genes in EHEs provides important diagnostic information, especially in cases with aberrant morphology or when biopsy material is limited. Moreover, clinicopathologic and research studies can be objectified based on these molecular fusion events, and biological and prognostic information will possibly influence therapeutic approaches.

## Abbreviations

EHE: Epithelioid hemangioendothelioma; PM-H: Pseudomyogenic hemangioendothelioma; ES: Epithelioid sarcoma.

## Competing interests

The authors declare that they no competing interests.

## Authors’ contributions

UF, TM, PG designed the study. PG, MV, MA, GP carried out the experiments. UF, TM, PG, AM drafted the manuscript. RV, AS, CH, DC, NS, RR, JB, JG contributed cases and participitated in data analysis. All authors approved the final version of the manuscript.
